# Methods for Pain Assessment in Calves and Their Use for the Evaluation of Pain during Different Procedures—A Review

**DOI:** 10.3390/ani11051235

**Published:** 2021-04-25

**Authors:** Theresa Tschoner

**Affiliations:** Clinic for Ruminants with Ambulatory and Herd Health Services, Centre for Clinical Veterinary Medicine, LMU Munich, Sonnenstrasse 16, 85764 Oberschleissheim, Germany; t.tschoner@lmu.de; Tel.: +49-89-218078850

**Keywords:** welfare, pain management, calves, ethogram, substance P, cortisol

## Abstract

**Simple Summary:**

Pain recognition in calves is difficult. Pain is assessed either subjectively or objectively. Subjective pain assessment can be done using ethograms or pain scales, or by evaluation of changes of the facial expression due to pain. The problem with subjective pain assessment is that the evaluation of the amount of pain a calf is experiencing depends on the evaluation and the experience of the observer. Variables for the objective description of pain are assessment of biomarkers in the blood (e.g., cortisol or substance P), use of algometry to measure mechanical nociceptive thresholds, activity measurements by use of accelerometers and pedometers, use of infrared thermography, and the assessment of heart rate, heart rate variability, feed and water intake, or weight gain. Studies about pain recognition and pain management in calves mostly use more than one variable. Often a combination of subjective and objective measures is used to evaluate the level of pain calves are experiencing and to improve pain recognition.

**Abstract:**

The evaluation and assessment of the level of pain calves are experiencing is important, as the experience of pain (e.g., due to routine husbandry procedures) severely affects the welfare of calves. Studies about the recognition of pain in calves, and especially pain management during and after common procedures, such as castration, dehorning, and disbudding, have been published. This narrative review discusses and summarizes the existing literature about methods for pain assessment in calves. First, it deals with the definition of pain and the challenges associated with the recognition of pain in calves. Then it proceeds to outline the different options and methods for subjective and objective pain assessment in calves, as described in the literature. Research data show that there are several tools suitable for the assessment of pain in calves, at least for research purposes. Finally, it concludes that for research purposes, various variables for the assessment of pain in calves are used in combination. However, there is no variable which can be used solely for the exclusive assessment of pain in calves. Also, further research is needed to describe biomarkers or variables which are easily accessible in the field practice.

## 1. Introduction

### 1.1. Definition of Pain

Molony [1] stated that “*animal pain is an aversive sensory and emotional experience representing an awareness by the animal of danger of threat to the integrity of its tissues; (note, there may not be any damage) it changes the animal’s physiology and behaviour to reduce or avoid damage, to reduce the likelihood of recurrence and to promote surgery*”. It is reasonable to think that mammals experience painful events in a similar way as humans do, as the neural pathways of pain sensation are similar [2]. There is no scientific definition of pain in animals which is universally accepted [3].

### 1.2. Challenges Associated with Recognizing Pain in Calves

Cattle are prey animals and do not show obvious pain behavior [2]; these animals try not to be noticed by predator animals if they are impaired, hurt, or in pain. This character of masking signs of pain to not express their weakness resulted in the impression that cattle are insensitive to pain [2,4,5]. It is a challenge to recognize and evaluate pain in cattle [5,6], making pain and pain management a major welfare problem in this species [7,8,9]. To be able to provide pain relief and analgesics, pain has to be recognized either by prescribing veterinarians or producers [6,9,10].

Pain studies conducted on adult cattle mostly concentrate on pain assessment during different conditions such as lameness [11,12] or mastitis [13]. In calves, pain studies largely focus on routine husbandry procedures, such as castration [14,15], disbudding [16,17], or dehorning [18,19], and the effect of analgesics on the experience of pain during and after these procedures [18,20]. Therefore, pain studies conducted in adult cattle are not very useful in relation to pain in calves.

There still are differences in the evaluation of the pain level calves are experiencing during and after different procedures. An important part of ensuring good welfare in calves is to accurately assess distress and pain [21]. Therefore, it is critical to define objective biomarkers and variables to evaluate pain in calves. The objective of this paper is to summarize and compare the different subjective and objective methods for pain assessment most commonly used in calves, and to outline the advantages and disadvantages of these pain measurement systems to provide researchers with the methods most suitable for their studies, and veterinarians with the knowledge how to evaluate pain in calves.

## 2. Pain Assessment in Calves

In 1995, Molony and his colleagues [22] stated that the assessment of pain in calves is difficult, as methods for calves have not been well developed. Nowadays, a number of tools for the recognition of pain is available, especially for research projects [9] (Table A1).

### 2.1. Subjective Pain Assessment

#### 2.1.1. Ethogram

An ethogram is a description of an array of behaviors which a particular species of animal is capable of showing over a defined period of time [23,24]. Behavior and changes in behavior can be used to assess pain in calves. State behaviors (such as postural behaviors) are suited for proportional documentation, while event behaviors (such as ear flicking) are best documented by numerical recording [24].

Ethograms can give very accurate indications of changes in the behavior of an animal over a larger period of time and are a powerful tool to document behavioral changes in conscious animals. However, large numbers of animals are required due to variability in behavior of animals [24].

Numerous studies have been published using ethograms in calves undergoing a painful stimulus, especially in relation to castration [14,22,25,26,27], disbudding [28], and dehorning [29,30,31].

There are several problems with using ethograms. The behavior can be affected, even substantially, by a person watching the animals [23]. In previous studies, assessment of behavior was done by direct observation, with different intervals of time between observations [22,29,31]. Recent studies work with video cameras on poles, to not influence the animals’ behavior, and provide continuous recordings [28,32,33]. Manual collection of data is labor intensive [34], and the analysis of video footage is time intensive. For the analysis of recordings, especially designed softwares [27,32] can be used.

The definitions of behavior recorded and measured should be clear [23]. Judgment of different behavior becomes more reliable and repeatable if the observer is appropriately trained and experienced. The approach to pain assessment should be consistent, to make sure that the same physiological and behavioral patterns are assessed in the individual animals [2]. All observers should be trained by the same, experienced person [35], and control of inter-observer reliability is needed [23,27,32]. Possibility of bias (unintentional or deliberate) should be considered [23]. Observers should be ignorant of the treatment at the time of observation and evaluation of behavior [23,27,29,30,32].

Different behaviors can be measured automatically nowadays, e.g., activity by use of accelerometers [27,35,36] or pedometers [37], or milk intake by use of automated calf feeding systems [28,36,38].

#### 2.1.2. Visual Analogue Scale

The Visual Analogue Scale (VAS) is a 100 mm horizontal line with a description of pain limits (0, “no pain” on the left and 10, “worst pain imaginable” on the right) at either end of the scale [39,40]. By use of a millimeter scale, this score provides 101 levels of pain intensity [40].

In veterinary medicine, observers place a mark along the line representing the amount of pain an animal is showing [32,33,35]. The distance either from the start of the line to the mark [32,33] or from the end point to the mark [35] is measured and used as an indicator of the animal’s pain response to a procedure. The VAS is reproducible, feasible, and sensitive [39]. Scoring can be performed based on the presence or absence of defined behaviors [35], by more than one observer [32,33,35]. Experienced animal scientists are needed [35,39], as results become more repeatable and reliable with training and experience [2]. As mentioned for the ethogram, a consistent approach to the assessment of pain is important, to ensure that an observer assesses the same physiological and behavioral signs in each animal [2].

In several surveys about pain assessment in calves, researchers presented a range of conditions in calves to be assessed using the VAS [41,42,43]. In other studies, the VAS was used to evaluate pain in calves after castration [32,33,35] or disbudding [44].

#### 2.1.3. Numerical Rating Scale

The Numerical Rating Scale (NRS) is a scale for pain assessment with two end points, “no pain” and “worst pain” or “pain as bad as it could be”. In humans, the NRS can be delivered either graphically or verbally and consists of an 11, 21 or 101 point scale [40]. In bovine medicine, the NRS mostly ranges from 0 (no pain) to 10 (worst pain imaginable) [7,45,46] or from 1 (no pain) to 10 (worst pain imaginable) [9,41,47], with no detailed definition of the category “worst pain imaginable”.

The NRS is mostly used in surveys evaluating pain assessment in adult cattle and calves. Several such studies have been published in the last 15 years [7,9,41,45,46,47].

Pain scores awarded for defined conditions and procedures in calves are similar among veterinarians (Table 1); however, authors use different categories for the NRS, which makes the direct comparison of pain scoring between studies impossible.

Problems with the NRS are lack of sensitivity (categories for a level of activity are often simplified) [48] and sensitivity [39]. The major problem with the NRS is the observer him- or herself. The evaluation of the amount of pain an animal experiences depends on the observer’s own experience and opinion [2]. As it has been shown that e.g., empathic veterinarians score pain in cattle higher than their less empathic colleagues [49], as do female veterinarians compared with male ones [9,41,45,46], the NRS, same as the VAS and other subjective methods for pain rating, presents some disadvantages.

#### 2.1.4. Facial Grimace Scales

In human beings, changes of facial expression are well studied [50]. In animals, facial expression has not been extensively studied. However, the facial expression of animals might represent an indicator for pain recognition [51].

**Figure 1:**
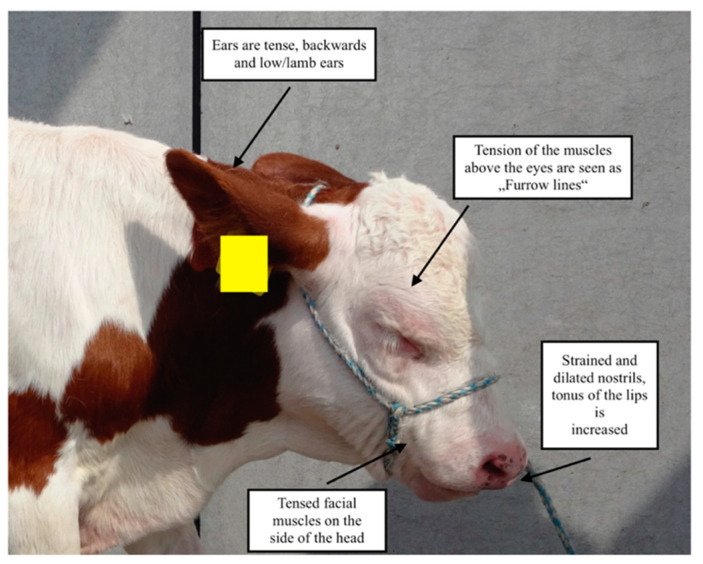
Image of the “bovine pain face” as described by Gleerup et al. (2015) [6], in a calf diagnosed with polyarthritis. The facial expression undergoes different changes due to pain. Note the position of the ears, the tension of the facial muscles and muscles above the eyes, and the dilated nostrils. The picture was taken by the author at the Clinic for Ruminants with Amulatory and Herd Health Services, LMU Munich.

In 2015, a Cow Pain Scale was described for pain evaluation in adult cattle; this included the “bovine pain face”. The features of the bovine pain face were evaluated in changes of four areas of the face (ears, eyes, facial muscles, and muzzle) [6] as described for the “Equine Pain Face” [52] by information gained from six healthy cows following a rumen fistulation surgery; facial expression was documented prior and after analgesic treatment [6]. Facial expressions were significantly different between cows not in pain compared with animals in pain [6]. Features of the pain face have been used for pain assessment in adult cattle following different procedures [8,51,53]. In calves, the use of the characteristics of the pain face or pain ears has been first described in 2017 [54]. Rääf and Olsen [54] stated that they used the description of the cow’s pain face and directly transferred these changes to calves’ facial expression, as calves’ facial muscles may already be fully developed [54]. An example of the bovine pain face in a calf is presented in Figure 1.

The facial expression should always be evaluated when the animal is undisturbed [6,54]. If animals are not used to handling, responses to human approach will be affected, therefore reducing the sensitivity of the facial grimace scale. Also, disease patterns, prevalence of acute or chronic pain, and age might influence the sensitivity and specificity of the bovine pain face [6].

Evaluation of the bovine pain face seems to be a promising tool for pain assessment in calves, and further studies are needed to describe changes due to pain in the facial expression of calves.

### 2.2. Objective Pain Assessment

#### 2.2.1. Cortisol

In newborn calves, cortisol is the major product secreted from the adrenal cortex: corticosterone only appears with the tenth day of life [55]. Cortisol concentrations show a circadian rhythm as a result of sleep and activity patterns superimposing the negative feedback control system [56]. Highest concentrations in the blood can be measured in the early morning hours [55] and lowest concentrations at night [56]. Cortisol concentrations in cattle are measured in the blood [14,22,57], faeces [58], saliva [57,59], milk [60], or hair [61]. Taking blood or saliva samples for the determination of cortisol is invasive [62]. The contamination with food due to feed or water intake, as well as increased salivation can interfere with saliva sampling. Hair samples are used as a retrospective marker of stress over longer time periods [63]. The evaluation of cortisol from the claw horn of calves has been described [64].

In calves, measurement of cortisol concentrations is mostly done from blood [14,35,65], either from plasma [17] or serum [18]. For analysis of cortisol concentrations, solid-phase competitive chemiluminescent enzyme immunoassays [18,66] or commercial radioimmunoassay kits [16,17] can be used. Performance of radioimmunoassay kits and chemiluminescent enzyme immunoassay methods are comparable in animal samples [67], with radioimmunoassays being the gold standard [68,69]. However, disadvantages for radioimmunoassays are the short shelf-life of the radioactive agents, the radiation safety hazards, and the strict requirements for waste disposal [68], whereas chemiluminescence offers stable reagents and hormone-enzymes and no toxic effects of the reagents [70].

Cortisol is the predominant indicator for (pain related) distress in calves. Cortisol has been used extensively as a research tool in pain research in calves, especially for pain studies for castration [14,15,22,35,71] and disbudding/dehorning [31,65,72,73].

The major disadvantage with using cortisol concentrations to assess pain in calves is that cortisol is also described to be a reliable indicator for acute stress. Cortisol concentrations change when an animal is experiencing stress; stress results in an immediate response of glucocorticoids, and an increase of cortisol concentrations within minutes. Concentrations reach levels which are several-fold greater than the baseline levels. The response of the glucocorticoids is proportional to the severity of the stress experienced [56]. Even if this includes acutely painful events, human presence or restraint can also influence cortisol concentrations [17]. The increase of cortisol concentrations in calves is dependent on the stressor [16] as well as on the individual and anxiety-related behavior of the animal [74]. Management techniques and external environmental factors can influence the rhythm cycle of cortisol [75]. Therefore, cortisol concentrations should best be evaluated in combination with substance P concentrations, to better differentiate between acute stress caused by handling, and distress caused by nociception [14].

#### 2.2.2. Substance P

In 2008, Coetzee et al. [14] compared plasma substance P (SP) and cortisol concentrations in bull calves undergoing surgical or stimulated castration. Whereas cortisol concentrations did not differ between groups, SP concentrations were significantly higher in the surgically castrated than the sham castrated animals for all time points following the procedure. It was suggested that with the determination of plasma concentrations of SP and cortisol, it might be possible to differentiate between distress associated with nociception and acute stress due to handling [14].

SP concentrations in cattle are either measured in the blood plasma [14,76], or via saliva samples [77]. Determination of SP concentrations is not an everyday procedure for the field practice, as blood tubes need to be spiked with a protease inhibitor (either benzadmidine [18,78] or aprotonin [76]) to stabilize SP. If processed immediately, SP concentrations do not differ among enzyme inhibitor treatments, but aprotonin seems to be the most effective inhibitor [79]. To prevent SP degradation, samples need to be kept on ice and plasma needs to be harvested after centrifugation within 1 [79] or 2 [76] hours after sampling. Analysis is done in the laboratory, e.g., by use of competitive immunoassay kits [14] or ELISA kits [76], which need to be validated for bovine plasma.

In the last ten years, SP has been used as a variable to assess pain during castration [14,32,33,78], dehorning/disbudding [17,18,65,80], and umbilical surgery [76]. Results of these studies imply that SP might be a promising biomarker to assess pain in calves. However, some studies investigating the relationship between painful procedures and SP concentrations report varying results [17], showing that SP concentrations did not differ between control calves and calves treated with analgesics for disbudding [65], or surgical castration [15]. There also seems to be an age difference in SP concentrations in calves [78]. Also, there are large inter-individual variations in SP concentrations [14,76].

Using SP as a biomarker for pain has some limitations, as SP is also involved in the activation of the immune system, the chemotaxis of neutrophil and eosinophil granulocytes, and the migration of cells of the immune system to inflamed tissues [81,82]. Human patients suffering from phobia, stress disorders and depression show an impaired transmission of SP [83,84]. Pregnant mice that are stressed release SP into the uterine tissue [85,86]. Therefore, it is possible that both stress as well as a state of inflammation result in a change of the SP concentrations in the plasma. It was suggested that SP and cortisol should be assayed in combination, so as to determine if the stressor resulting in the release of SP is caused by pain or (restraint) stress [17].

In conclusion, SP, in combination with cortisol, can be used to differentiate between distress caused by pain or stress caused by handling [14] and is a promising possibility to objectively evaluate pain in calves for research projects. However, basic research needs to be done about the role of inflammation or stress on SP concentrations in the plasma of calves.

#### 2.2.3. Mechanical Nociceptive Threshold

The mechanical nociceptive threshold (MNT) is defined as the amount of pressure (in kilogram of force) a calf tolerates over a defined area [87], measured with an algometer [18,87]. This test is semi-quantitative and is affected by the behavior of the individual calf. Therefore, a baseline value should be evaluated and defined for each animal [88]. Several studies used MNT to measure pain sensitivity following cautery dehorning in calves. A pressure algometer is equipped with a round rubber tip (diameter of 1 cm) [18,44,65,87,89]. After habituation of the calf to the examiner’s touch, the examiner’s hand is replaced with the algometer; the rubber tip is placed directly beside the horn bud, covering the cautery wound and the edge of the normal tissue. Pressure is applied (1 kgf per second) until the calf withdraws its head [18,87]. In other studies, force was applied at an approximate rate of 2 N/s to the skin surface of disbudded calves, only withdrawing the algometer if the calves showed signs of discomfort or the applied force had reached a cutoff [44,89]. Pressure can be applied to 2 [65] or 4 sites per horn [18,28,87].

Heinrich et al. [87] stated that the decrease in MNT, as seen in dehorned calves, is caused by pain and that pressure algometry can be used to objectively assess pain in cattle. After cautery dehorning, tolerance to pressure decreased in calves [18,65]. Research shows that the administration of non-steroidal anti-inflammatory drugs result in an increase of MNT following dehorning [17,18,65,87], as did the application of a cornual block prior to caustic paste disbudding [28].

The advantage of pressure algometry is its easy use in clinical settings, as well as the low costs [90]. Repeatability of pain thresholds measured with MNT is good, and values are stable [89]. However, there is no gold standard, and investigators need to be practiced to reliably use MNT [44]. Intra- and inter-observer variability needs to be considered [90]. Research in dogs showed that tip diameter, position of the animal, and the anatomical site might affect the results of MNT; the individual animal had the most significant effect on the efficacy of MNT [91]. Also, pressure algometry only evaluates the local sensitivity, e.g., around the horn bud, and repeated handling might increase the avoidance response in calves [65], influencing study results.

#### 2.2.4. Activity

Accelerometers can objectively measure and evaluate movements and changes in an animal’s behavior [35], in cattle as well as in calves. The accuracy of 2-dimensional accelerometers is 98.3% for posture in calves [92].

A triaxial accelerometer is a sensor which returns a real valued estimate of the object’s acceleration along three axes (*x*-, *y*-, and *z*-axis), also enabling to estimate velocity and displacement [93]. Accelerometers are placed horizontally on one leg, with the *x*-axis pointing towards the dorsal plane of the calf [36]. Tri-axial accelerometers measure the angle of tilt to the earth, as well as the amount of dynamic acceleration, determining the position relative to the ground, and the direction and speed of movement [94]. Depending on the accelerometer, different sensor outputs, such as Standing, Lying, Walking, or Step counts [35,95] are included. Data from the accelerometers can be downloaded on computers using the producer’s software [35,36]. The G-force readings are then converted into binary values (such as 0 for lying, and 1 for standing), and summaries of activities are calculated using codes designed for that purpose [36]. The sum of each activity can be tabulated for each study period [35].

Accelerometers have been developed and validated to measure a wide variety of different behaviors in calves, in the form of pedometers, ear tags, or collars [94]. In the last decade, accelerometers have been used extensively for the assessment of activity in calves to assess changes to activity due to pain after different procedures [35,71,87,92,95,96].

Accelerometers are non-invasive [92]. Other advantages of these devices are their small size and weight, the low cost, and their ability to record behavioral data over a long period of time (days or months) in high-resolution quality [94]. Long time recording requires an adequate size of memory, which might increase the cost; however, this problem can be overcome by wireless collection of data, which restricts the area an animal is able to move in [97].

Also, as accelerometers are attached to the animal’s legs, they might influence the physiological behavior, and can possibly be removed by the animal itself [97].

In conclusion, accelerometers are a suitable tool for the assessment of pain in calves by measuring calves’ activity and changes thereof and have been described to likely be more sensitive than video analysis or direct behavioral observations of calves [87].

#### 2.2.5. Monitoring Steps

Recording of number of steps taken by calves after a painful procedure, such as castration, can be a useful measurement of postsurgical pain in calves [37]. Pedometers are used to objectively monitor and quantify the number of steps taken by calves [37,98] and can be fixed on either leg of a calf [20,37,88,99,100]. Pedometers contain an on-board algorithm to calculate the number of steps from the raw data [98]. The number of steps recorded on pedometers is within 5% of the actual number of steps taken by calves [37]. The range of pedometers to their base unit is about 400 m [101]. They are easily attached and used [98].As described for accelerometers, pedometers are small, non-invasive, and are not likely to influence the natural patterns of animal behavior [101,102]. Another advantage is the low cost of investment, as well as the low labor input [98].

Data collection with pedometers is described in numerous studies about pain assessment in calves undergoing painful procedures, such as castration [20,37,99].

Pedometers can also be used to assess the movement of the tail. Pedometers are fixed 10 cm from the tail base and read manually, defining the “steps” as tail movements, e.g., for pain assessment following castration [35]. The fact that pedometers directly measure the animal’s locomotion also makes them a valuable tool for assessing and monitoring musculoskeletal pain [98], such as lameness [100].

Stress, as well as pain, may influence the distance a calf travels. It has been published that calves took more steps for 3 days after weaning [103]. The number of steps calves take varies among days and due to environmental conditions [98]. Also, pain assessment with pedometers might be more suitable for some procedures (as castration) compared with others (dehorning). The pain calves experience after dehorning might not have as much of an impact on resting after the procedure, as does the pain associated with castration [101].

Pedometers can be used to record continuous and individual animal data, providing inferred behavioral data (objective data). However, use of pedometers requires further investigation, and should especially be analyzed in combination with ethograms [101].

#### 2.2.6. Measurement of Eye Temperature

In veterinary medicine, infrared thermography (IRT) is a term used to describe in vivo digital imaging of an animal by use of an infrared camera. The thermal imaging cameras show temperatures with a precision of 0.08 °C. Different temperatures are represented as various colors, and thermal maps of an object or a body surface can be interpreted with computers [104].

In the last years, IRT has been used for detection of inflammation, such as mastitis [105,106] or hoof lesions [11,107], as a non-invasive method to predict methane production and emission [108], and welfare evaluation [62] in bovine medicine.

In previous studies, eye (ocular) temperature was measured by use of infrared thermography cameras. Images were taken from a distance of 0.5 m at a right angle (90 °C). The maximum temperature (measured in °C) was recorded in the lower eyelid (medial posterior palpebral border) and the eye caruncle [109,110] at different intervals [109,111]. Research suggests that by measuring the temperature of the eye with IRT, changes of the sympathetic nervous system activity can be used for the study of pain recognition in calves [109,110,111]. A decrease in eye temperature following a painful stimulus, such a disbudding, can be attributed to sympathetically mediated alterations in the blood flow in the capillary beds of the eye, redirecting the blood flow to organs and skeletal musculature due to the “fight and flight” response [109,112]. Eye temperature decreased rapidly after disbudding but increased again from 5 min after the treatment and was higher for disbudded compared with control calves [109]. Similar research was done for pain assessment after castration [111]. These results imply that IRT of the eye might be used to assess pain in calves.

Eye temperature can be measured easily, and the location provides no interference of fur or hair. Orbital temperate is less variable than temperatures taken from other areas [113]. Even if the use of IRT is simple, non-contact and non-invasive [104], a high technical as well as financial input is required even if minimal standards of quantification is used [114]. The area which has to be measured needs to be defined and analyzed in a standardized way, because automated analysis through a computer-based algorithm is only feasible within a standardized framework [114]. There is little evidence concerning the physiological changes of skin temperature in cattle [104]. Also, eye temperature is not only influenced by pain, but also by stress [112,113] and disease [113]. To differentiate between pain and fear/stress, IRT should be used in combination with other variables for the assessment of pain, such as cortisol [17], substance P [15], or behavior and heart rate (variability) [115].

#### 2.2.7. Heart Rate and Heart Rate Variability

In calves, measurement of heart rate (HR) is done by auscultation in restrained animals [28], or by use of a girth heart rate recorder and data loggers [35]. Heart rate variability (HRV), or changes of the time interval between heartbeats, can be used to evaluate the activity of the sympathetic nervous system as part of the autonomic nervous system [111,116], and the autonomic nervous signs associated with stress in cattle [117], representing changes in the balance between sympathetic and parasympathetic branches of the autonomic nervous system [118]. Changes in the sympathetic and vagal balance occur due to psychological and environmental stressors, but also related to diseases [116].

HRV is assessed by use of a fitting heart rate monitor fixated with a belt to the clipped skin behind the left foreleg of the animal, which records nonlinear measures [111,117,119]. The simplest way to measure HRV is by time domain measures. These can be divided into two classes: (1) the measurement of variability from interbeat intervals (IBIs) which is the easiest, but least informative way to calculate IBIs and HR, and (2) the measurement of variability acquired from differences between IBI. For these, the best parameter is the root mean square of successive differences (RMSSD), which is determined by calculation of the difference between consecutive IBIs. The IBIs are squared and summed, the values averaged, and the square root is obtained [116]. Sequences of IBIs are converted into geometrical forms; afterwards, assessment of HRV can be extracted from these forms [116]. These measures can be quantified using available software [117].

Research showed that HR is significantly increased in animals experiencing pain, such as castration [35,111] or disbudding [117]; HRV changes following a painful event [111]. According to these studies, the measurement of heart rate can be used to assess if calves are in a painful state, especially an acute one [111]. However, several studies showed that HR and HRV, if measured over short-term periods of time, return to baseline rapidly [111,116].

Not only pain, but also other factors result in changes of HR and HRV. In humans as well as in animals, HRV is influenced by sex, age, respiration, fitness, posture, physical activity, and diurnal rhythms [116]. External stress as well as internal stress results in significantly higher heart rates in calves, and the RMSSD of R-R-interval decreased significantly with increased stress load, due to reduction of the vagal tone [119]. Changes in cardiac activity have also been observed in animals in an anticipatory manner prior to the expression of alterations [116].

The major advantage of monitoring HR and HRV is that this method is non-invasive [111,116], except if implantable devices are used [116]. Depending on the equipment, systems used for the assessment of HRV are expensive; an alternative which is affordable is the use of commercially available monitors which detect R-peaks of the electrocardiogram, storing IBI in digital form [116]. To avoid misinterpretation of data, the identification of artefacts or ectopic beats in IBI data is important [116].

Bearing in mind that not only pain but also stress and other factors result in changes of HRV [111,116,117], it is advisable to evaluate HR and HRV in combination with other methods for pain assessment, such as cortisol, SP, IRT, or behavior [35,65,111,115].

#### 2.2.8. Feed Intake

Feed intake serves as an indicator for cattle welfare and well-being. Halters including a pressure sensor integrated into the noseband record the jaw movements during chewing and ruminating in adult cattle [120,121], and during rumination in calves [122]. In calves, monitoring of feed intake can be done either by directly weighting the difference between offered and consumed feedstuff [87], or by analyzing records of automatic calf feeding systems (ACFS) [28,36,38]. For example, radio-frequency ear tags connected to a feed bunk monitoring system inside the pens can be used to record the feeding behavior of calves. The electronic monitoring system displays various parameters [28].

ACFS need to be accurate and precise in their quantity for feed delivery. Calibration following the manufacturer’s recommendations is necessary. Until now, there is no validation of the accuracy and precision of ACFS under different on-farm circumstances or at farm level [94].

Feed intake has been used as an indicator for pain in calves in several studies [28,38,87]. However, feed intake also changes before [36,94] and during [123] clinical disease, and due to stress [36]. Therefore, recording feed and milk intake seems to be more suitable for detecting diseases, than for directly assessing pain in calves. Therefore, recording and analysis of feed intake should not be used as the single indicator for pain in calves, but in combination with other variables for the assessment of pain.

#### 2.2.9. Average Daily Weight Gain

Measurement of body weight and average daily weight gain (ADG) is used as an objective assessment for the evaluation of post-operative pain in calves [101]. Calves showed a reduction of weight after surgical castration [27,124]. The weight reduction could indicate the experience of chronic stress, which could not be detected with other physiological variables, such as salivary cortisol or SP [27]. It is also possible that differences in growth are related to the hormonal status of the control group, age at castration, and length of the study period after castration [124].

Also, other studies found no effect of the use of analgesia on the ADG of calves for cautery dehorning [18] or disbudding [65], following (caustic paste) disbudding [17], or band-castration [125]. Branding did not influence the ADG [126].

Therefore, the measurement of ADG might not be particularly useful for the assessment of pain in calves, and as the ADG is also influenced by (chronic) stress [27], more suitable biomarkers and variables for the measurement of pain should be used to assess pain in calves.

## 3. Conclusions

There are a number of tools available to assess pain in calves. Subjective pain assessment heavily relies on the experience and evaluation of the observer, representing only his or her subjective assessment, and is the method practicing veterinarians most heavily rely on in their field work. As the use of analgesics in calves mostly relies on the perception and recognition of pain by the attending veterinarian, further training in the subjective assessment of pain should be focused on.

Objective pain assessment is mostly done by use of biomarkers in the blood plasma and serum but can also be done by analysis of other variables. As some of these variables are not only changed due to acute pain, but also other factors such as stress, restraint, or management, none of these variables can be used solely for the objective assessment of pain in calves. Therefore, it is advisable to use a combination of objective and subjective variables for research purposes, to best evaluate the severity of pain an animal is experiencing. Further studies concentrating on the description of an objective variable to exclusively assess pain in calves are needed. The more objective variables used to evaluate pain in calves are, the less are needed.

In the course of precision livestock farming, objective variables for the detection of pain, such as accelerometers, automated feeding systems, evaluation of IRT, or automated detection of the pain face, should be further developed.

To improve the recognition of pain in calves, research about the development of tools for the assessment of pain should focus more on the availability and practicability of these tools in the field. Tools for pain assessment should be easily accessible both for veterinarians and producers, to make pain recognition easier, and thereby improving analgesic treatment and pain management in calves.

## Figures and Tables

**Table 1:** Median Pain Scores (including range) as awarded by veterinarians for different procedures and conditions in calves, assuming no analgesic drugs were administered. Numerical rating scores (NRS) differed between studies. Total number (n) of respondents to the surveys is given in brackets.

Conditions	Authors (Year) [Reference]					
	Huxley et al. ^†^ (2006) [7]	Laven et al. ^†^ (2009) [45]	Kielland et al. ^‡,^* (2009) [41]	Weber et al. ^‡^ (2012) [47]	Remnant et al. ^‡^ (2017) [9]	Tschoner et al. ^†^ (2020) [46]
	(*n* = 615)	(*n* = 166)	(*n* = 89)	(*n* = 741)	(*n* = 242)	(*n* = 274)
Distal limb/long bone fracture	8 (2–10)	9 (2–10) ^♦^	8 (4–10)	8 (2–10)	9 (5–10)	8 (3–10)
Following dystocia	4 (1–10)	4 (1–10)	4 (1–8)	5 (1–10)	5 (1–9)	5 (0–10)
Umbilical abscess	5 (1–10)	5 (1–9)	n.A.^1^	6 (1–10)	6 (2–9)	n.A. ^1^
Umbilical infection	n.A.^1^	n.A.^1^	5 (2–8)	n.A.^1^	n.A. ^1^	7 (2–9)
Joint ill	7 (1–10)	8 (2–10)	7 (3–9)	7 (2–10)	7 (3–10)	8 (1–10)
Pneumonia	6 (1–10)	6 (2–10)	6 (2–10)	6 (1–10)	7 (1–10)	6 (1–10)
Intestinal Ill/Enteritis	n.A. ^1^	n.A. ^1^	6 (1–10)	n.A. ^1^	n.A. ^1^	5 (1–10)
Procedures						
Castration (surgical)	6 (2–10)	8 (2–10)	n.A. ^1^	10 (2–10)	7 (2–10)	9 (1–10)
Castration (rubber ring)	6 (1–10)	5 (2–10)	n.A. ^1^	n.A. ^1^	6 (2–10)	n.A. ^1^
Castration (Burdizzo)	7 (2–10)	6 (2–10)	n.A. ^1^	8 (1–10)	7 (2–10)	9 (2–10)
Umbilical hernia surgery	8 (2–10)	8 (3–10)	n.A. ^1^	9 (2–10)	n.A. ^1^	9 (1–10)
Disbudding	7 (2–10)	8 (3–10)	n.A. ^1^	n.A. ^1^	7 (2–10)	n.A. ^1^
Dehorning	n.A. ^1^		n.A. ^1^	8 (1–10)	n.A. ^1^	8 (1–10)

**^†^** Pain scoring by use of an NRS ranging from 0 (no pain) to 10 (worst pain imaginable); **^‡^** Pain scoring by use of an NRS ranging from 1 (no pain) to 10 (worst pain imaginable). * Survey conducted among veterinary students. ^♦^ Pain assessment for “Repair of distal limb fracture”. ^1^ n.A. Not Asked.

## Data Availability

Data is contained within the article.

## Appendix A

**Table A1:** Overview of tools used for the assessment of pain in calves in research literature. Study groups includes the age or the weight of the calves used for the trials. Tools used for the pain assessment are given as Outcome Parameter. Observation of behavior was defined as Ethogram. References (**Ref.**) are given in the right column.

Year	Author	Study Population	Procedure	Outcome Parameter	Assessment	Ref.
1995	Molony et al.	2 to 3 days (Dairy)	Castration (Burdizzo and/or rubber ring)	Cortisol Behavior	Blood plasma Ethogram	[22]
	Morisse et al.	4 to 6 weeks (Dairy)	Dehorning (Chemical and heat cauterization)	Cortisol Activity Behavior	Blood plasma Video recording Ethogram	[72]
1997	Schwartzkopf-Genswein et al.	303 ± 2 kg (Beef-cross)	Branding (Freeze or Hot-Iron)	Body Weight	Scale	[126]
1998	McMeekan et al.	3 to 4 months (Dairy)	Dehorning (Scoop)	Cortisol	Blood plasma	[127]
2000	Faulkner and Weary	4 to 8 weeks (Dairy)	Dehorning (Hot-Iron)	Behavior	Ethogram	[29]
2002	Eicher et al.	3 to 5 weeks (Dairy)	Tail docking	Behavior	Ethogram (Video)	[128]
	Sutherland et al.	3 to 4 months (Dairy)	Dehorning (Scoop)	Cortisol	Blood plasma	[129]
2005	Schwartzkopf-Genswein et al.	26 to 59 days and 54 to 80 days (Dairy)	Dehorning, Castration (Surgical, 21 days later)	Cortisol Heart rate Behavior	Blood plasma Heart rate monitor Ethogram	[25]
	Vickers et al.	10 to 35 days (Dairy)	Dehorning (Hot-Iron)	Behavior	Ethogram (Video)	[30]
2008	Coetzee et al.	4 to 6 months (Beef-cross)	Castration (Surgical)	Cortisol Substance P Behavior	Blood plasma Blood plasma Ethogram (Scores)	[14]
	Stilwell et al.	5 to 6 months (Dairy)	Castration (Clamp)	Cortisol Behavior	Blood plasma Ethogram	[26]
	Stilwell et al.	10 to 40 days (Dairy)	Dehorning (Caustic paste)	Cortisol Behavior	Blood plasma Ethogram	[31]
	White et al.	278.1 ± 25.6 kg (Beef)	Castration (Surgical)	Posture Behavior (Activity)	Accelerometer Accelerometer	[92]
2009	Currah et al.	3 months (Beef-cross)	Castration (Surgical)	Behavior (Activity) Vocalization Force of struggling Stride length Posture and locomotion Behavior activity	Pedometer Recording MMD ^1^ Video recording Visual assessment Live observation	[37]
	Heinrich et al.	6 to 12 weeks (Dairy)	Dehorning (Cautery)	Cortisol Heart rate Respiratory rate	Blood serum Auscultation Counting	[73]
	Stewart et al.	33 ± 0.3 days (Dairy)	Dehorning (Hot-Iron)	Ocular temperature Heart rate/HRV ^2^	IRT ^3^ Heart rate monitor	[110]
2010	Coetzee et al.	4 to 6 months (Beef-cross)	Castration (Surgical)	Cortisol Behavior	Blood serum Ethogram	[66]
	Heinrich et al.	6 to 12 weeks (Dairy)	Dehorning (Cautery)	MNT ^4^ Behavior Activity Feed/water intake	Algometry Ethogram (Video) Accelerometer Scale	[87]
	Stewart et al.	4 months (Dairy)	Castration (Surgical)	Cortisol Catecholamines Ocular temperature Heart rate/HRV	Blood plasma Blood plasma IRT Heart rate monitor	[111]
	Stilwell et al.	37 ± 4 days (Dairy)	Disbudding (Hot-Iron)	Cortisol Behavior	Blood plasma Ethogram	[16]
2012	Coetzee et al.	Not available.	Dehorning (Scoop)	Cortisol Substance P Activity Heart Rate Average Daily Gain	Blood serum Blood plasma Accelerometer Auscultation Scale	[80]
	Pauly et al.	16 to 20 weeks (Dairy)	Castration (Surgical) Dehorning (Hot-Iron) (Concurrent)	Activity	Accelerometer	[95]
	Theurer et al.	10 weeks Not available	Dehorning (Hot-Iron Thermocautery)	Activity	Accelerometer	[96]
2013	Allen et al.	8 to 10 weeks (Dairy)	Dehorning (Hot-Iron)	Cortisol Substance P MNT Ocular Temperature Body Weight	Blood serum Blood plasma Algometry IFR ^3^ Weighting	[18]
	Dockweiler et al.	8 weeks and 6 months (Dairy)	Castration (Band, Cut-and-clamp, Cut-and-Pull)	Cortisol Substance P Heart rate/HRV Ocular Temperature Electroencephalogram Electrodermal Activity	Blood serum Blood plasma Heart rate monitor IRT Electrodes Electrodes	[78]
	Mintline et al.	4 to 4.5 weeks (Dairy)	Disbudding (Hot-Iron)	Behavior Wound sensitivity	Ethogram (Video) Von Frey Monofilaments	[130]
	Pieler et al.	56 ± 3 days (Dual-purpose)	Castration (Partial Scrotal Resection, Orchidectomy, Burdizzo)	Cortisol Behavior Locomotion Heart rate/HRV	Saliva Ethogram Pedometer Heart rate monitor	[99]
	Repenning et al.	309.8 ± 59.04 and 300.8 ± 4.96 (Beef)	Castration (Band)	Substance P Behavior Body Weight	Blood plasma Ethogram (Video) Scale	[125]
2014	Coetzee et al.	4 to 6 months (Beef)	Induced Lameness	Cortisol Lameness Score Activity Weight Gain	Blood serum Visual scoring Pedometer Scale	[100]
	Coetzee et al.	2 months (Dairy)	Castration (Surgical)	Cortisol Behavior Heart Rate Respiratory Rate Activity	Blood Serum Ethogram (Scores) Auscultation Auscultation Pedometer	[20]
2015	Bates et al.	3 to 6 weeks (Dairy-cross)	Disbudding (Gas-dehorner)	Milk intake Body weight	ACFS ^5^ Scale	[38]
	Stock et al.	4 to 6 weeks (Dairy)	Disbudding (Cautery)	Cortisol Substance P Ocular temperature Heart Rate MNT Average Daily Gain	Blood plasma Blood plasma IRT Auscultation Algometry Scale	[65]
2016	McCarthy et al.	3 months (Beef)	Castration (Surgical)	Cortisol	Blood plasma	[131]
	Kleinhenz et al.	6 to 8 weeks (Dairy)	Dehorning (Electrocautery)	Cortisol Substance P Ocular/skin temperature MNT	Blood plasma Blood plasma IRT Algometry	[19]
	Olson et al.	4 to 5 months (Dairy)	Castration (Band, Surgical)	Cortisol Substance P Heart rate Activity Behavior Tail movement	Blood plasma Blood plasma Girth heart rate recorder Accelerometer Ethogram Tail pedometers	[35]
2017	Marti et al.	1 weeks and 2 months And 4 months (Beef-cross)	Castration (Band, Surgical)	Cortisol Substance P Scrotal temperature Behavior Weight Gain Activity Stride length	Saliva and Hair Blood plasma IRT Ethogram (Video) Scale Accelerometer Video recording	[27]
	Meléndez et al.	7 to 8 months (Beef-cross)	Castration (Surgical)	Cortisol Substance P Behavior Activity Feeding Behavior Weight Gain	Saliva and Hair Blood serum Ethogram (Video) Accelerometer Radio-frequency ear tag Scale	[32]
	Musk et al.	6 to 8 months (*Bos indicus*)	Castration (Surgical)	Bodyweight Activity	Scale Pedometer	[101]
	Winder et al.	Not available (Dairy)	Dehorning (Caustic Paste)	MNT Behavior Heart rate Respiratory rate Feeding behavior Standing/Lying bouts Play behavior	Algometry Ethogram Auscultation Observation of flank ACFS Data loggers Visual observation	[28]
2018	Adcock et al.	3 or 35 days (Dairy)	Disbudding (Hot-Iron)	MNT Skin temperature Weight gain	Algometry Thermography Scale	[132]
	Kleinhenz et al.	9 months (Dairy)	Castration (Surgical)	Cortisol Substance P Ocular temperature Gait analysis	Blood plasma Blood plasma IRT Mat-based pressure/force measurement system	[15]
	Meléndez et al.	7 to 8 days (Beef-cross)	Castration (Band, Surgical)	Cortisol Substance P Scrotal temperature Body Weight Behavior Stride length Standing/Lying Bouts	Saliva Blood plasma IRT Scale Ethogram (Video) Video recording Data logger	[33]
	Mirra et al.	1 and 4 weeks (Dairy)	Disbudding (Cautery)	Cortisol Heart Rate Respiratory rate Tactile sensitivity score MNT Behavior Multidimensional pain scale	Blood plasma Auscultation Counting Von Frey filaments Algometry Ethogram (Video) VAS ^6^	[44]
	Park et al.	6,3 ± 0,09 months (Beef)	Castration (Surgical)	Cortisol Substance P	Blood plasma Blood plasma	[133]
	Sutherland et al.	27 ± 1,5 days (Dairy)	Disbudding (Cautery)	Feeding Behavior Lying Behavior	ACFS Accelerometer	[36]
	Tschoner et al.	38 ± 9 days (Dual-purpose)	Umbilical surgery	Substance P	Blood plasma	[76]
2019	Byrd et al.	4 to 7 weeks (Dairy)	Disbudding (Gas-dehorner)	Heart Rate/HRV Behavior	Heart rate monitor Ethogram (Video)	[117]
	Casoni et al.	7 and 28 days (Dairy)	Disbudding (Electric cautery)	MNT Mechanical allodynia score Nociceptive reflexes	Algometry Von Frey Filaments Electrical/Laser stimulation	[89]
	Cuttance at al.	2 to 6 weeks (Dairy)	Disbudding (Thermocautery)	Behavior MNT Weight Gain	Ethogram (Video) Algometry Scale	[134]
	Karlen et al.	41.2 ± 0.1 kg (Dairy)	Disbudding (Caustic paste)	Cortisol Substance P Eye/Skin temperature MNT Average Daily Weight	Blood plasma Blood plasma IRT Algometry Scale	[17]
	Jimenez et al.	33 to 54 days (Dairy)	Cornual nerve block	Heart rate/HRV Eye temperature Behavior	Heart rate monitor IRT Ethogram (Video)	[115]
2020	Adcock et al.	24 to 38 days (Dairy)	Disbudding (Cautery)	Behavior Pain sensitivity	Ethogram (Video) Pin Prick Test	[135]
	Adcock and Tucker	21 to 28 days (Cautery)	Disbudding (Cautery)	Behavior Wound sensitivity	Ethogram (Video) Algometry	[136]

^1^ Movement Measuring Device, ^2^ Heart Rate Variability, ^3^ Infrared Thermography, ^4^ Mechanical Nociceptive Threshold, ^5^ Automated Calf Feading System, ^6^ Visual Analogue Scale.

## References

[1] Molony V. (1996). Comments on Anand and Craig. Pain.

[2] Hudson C., Whay H., Huxley J. (2008). Recognition and management of pain in cattle. Practice.

[3] Viñuela-Fernández I., Jones E., Welsh E.M., Fleetwood-Walker S.M. (2007). Pain mechanisms and their implication for the management of pain in farm and companion animals. Vet. J..

[4] O’Callaghan M.W. (2002). Lameness and associated pain in cattle-challenging traditional perceptions. Practice.

[5] McLennan K.M. (2018). Why Pain Is Still a Welfare Issue for Farm Animals, and How Facial Expression Could Be the Answer. Agriculture.

[6] Gleerup K.B., Andersen P.H., Munksgaard L., Forkman B. (2015). Pain evaluation in dairy cattle. Appl. Anim. Behav. Sci..

[7] Huxley J.N., Whay H.R. (2006). Current attitudes of cattle practitioners to pain and the use of analgesics in cattle. Vet. Rec..

[8] Feist M., Köstlin R., Nuss K. (2008). Claw surgery in cattle: The benefit of perioperative analgesics. Tierarzt. Prax. Ausg. G.

[9] Remnant J.G., Tremlett A., Huxley J.N., Hudson C.D. (2017). Clinical attitudes to pain and use of analgesia in cattle-Where are we 10-years on?. Vet. Rec..

[10] Wikman I., Hokkanen A.-H., Pastell M., Kauppinen T., Valros A., Hänninen L. (2016). Attitudes of beef producers to disbudding and perception of pain in cattle. Anim. Welf..

[11] Alsaaod M., Büscher W. (2012). Detection of hoof lesions using digital infrared thermography in dairy cows. J. Dairy Sci..

[12] Bustamante H.A., Rodríguez A.R., Herzberg D.E., Werner M.P. (2015). Stress and pain response after oligofructose induced-lameness in dairy heifers. J. Vet. Sci..

[13] Fitzpatrick C., Chapinal N., Petersson-Wolfe C., Devries T., Kelton D., Duffield T., Leslie K. (2013). The effect of meloxicam on pain sensitivity, rumination time, and clinical signs in dairy cows with endotoxin-induced clinical mastitis. J. Dairy Sci..

[14] Coetzee J.F., Lubbers B.V., Toerber S.E., Gehring R., Thomson D.U., White B.J., Apley M.D. (2008). Plasma concentrations of substance P and cortisol in beef calves after castration or simulated castration. Am. J. Vet. Res..

[15] Kleinhenz M., Van Engen N., Smith J., Gorden P., Ji J., Wang C., Perkins S., Coetzee J. (2018). The impact of transdermal flunixin meglumine on biomarkers of pain in calves when administered at the time of surgical castration without local anesthesia. Livest. Sci..

[16] Stilwell G., Carvalho R., Carolino N., Lima M., Broom D. (2010). Effect of hot-iron disbudding on behaviour and plasma cortisol of calves sedated with xylazine. Res. Vet. Sci..

[17] Karlen K.J., Baier F.S., Odegard S.L., Baumann R.M., Coetzee J.F., Kehoe S.I., Vogel K.D. (2019). Efficacy of oral meloxicam as primary pain mitigation following caustic paste disbudding of three day old Holstein calves. Transl. Anim. Sci..

[18] Allen K., Coetzee J., Edwards-Callaway L., Glynn H., Dockweiler J., KuKanich B., Lin H., Wang C., Fraccaro E., Jones M. (2013). The effect of timing of oral meloxicam administration on physiological responses in calves after cautery dehorning with local anesthesia. J. Dairy Sci..

[19] Kleinhenz M.D., Van Engen N.K., Gorden P.J., Coetzee J.F. (2016). Topical Flunixin Meglumine Effects on Pain Associated Biomarkers after Dehorning. Top. Flunixin Meglumine Effects Pain Assoc. Biomark. Dehorning.

[20] Coetzee J.F., Lechtenberg K.F., Stock M.L., KuKanich B. (2013). Pharmacokinetics and effect of intravenous nalbuphine in weaned Holstein calves after surgical castration. J. Vet. Pharmacol. Ther..

[21] Stilwell G.T. (2009). Pain Evaluation and Control after Routine Interventions in Cattle.

[22] Molony V., Kent J., Robertson I. (1995). Assessment of acute and chronic pain after different methods of castration of calves. Appl. Anim. Behav. Sci..

[23] Fraser A.F., Broom D.M., Fraser A.F., Broom D.M. (1990). Describing, recording and measuring behaviour. Farm Animal Behaviour and Welfare.

[24] Johnson C.B., Gibson T.J., Flint P., Wilson P.W., Mellor D.J. New techniques for pain recognition: What are the applications, where are the limits?. Proceedings of the Australian Anim Welf Strategy International Conference.

[25] Schwartzkopf-Genswein K.S., Booth-McLean M.E., McAllister T.A., Mears G.J. (2005). Physiological and behavioural changes in Holstein calves during and after dehorning or castration. Can. J. Anim. Sci..

[26] Stilwell G., Lima M.S., Broom D.M. (2008). Effects of nonsteroidal anti-inflammatory drugs on long-term pain in calves castrated by use of an external clamping technique following epidural anesthesia. Am. J. Vet. Res..

[27] Marti S., Meléndez D.M., Pajor E.A., Moya D., Heuston C.E.M., Gellatly D., Janzen E.D., Schwartzkopf-Genswein K.S. (2017). Effect of band and knife castration of beef calves on welfare indicators of pain at three relevant industry ages: II. Chronic pain. J. Anim. Sci..

[28] Winder C.B., Leblanc S.J., Haley D.B., Lissemore K.D., Godkin M.A., Duffield T.F. (2017). Clinical trial of local anesthetic protocols for acute pain associated with caustic paste disbudding in dairy calves. J. Dairy Sci..

[29] Faulkner P., Weary D. (2000). Reducing Pain After Dehorning in Dairy Calves. J. Dairy Sci..

[30] Vickers K., Niel L., Kiehlbauch L., Weary D. (2005). Calf Response to Caustic Paste and Hot-Iron Dehorning Using Sedation With and Without Local Anesthetic. J. Dairy Sci..

[31] Stilwell G., Lima M., Broom D. (2008). Comparing plasma cortisol and behaviour of calves dehorned with caustic paste after non-steroidal-anti-inflammatory analgesia. Livest. Sci..

[32] Meléndez D.M., Marti S., Pajor E.A., Moya D., Gellatly D., Janzen E.D., Schwartzkopf-Genswein K.S. (2017). Effect of timing of subcutaneous meloxicam administration on indicators of pain after knife castration of weaned calves. J. Anim. Sci..

[33] Meléndez D.M., Marti S., Pajor E.A., Moya D., Gellatly D., Janzen E.D., Schwartzkopf-Genswein K.S. (2018). Effect of a single dose of meloxicam prior to band or knife castration in 1-wk-old beef calves: I. Acute pain. J. Anim. Sci..

[34] Millman S.T. (2013). Behavioral Responses of Cattle to Pain and Implications for Diagnosis, Management, and Animal Welfare. Vet. Clin. North Am. Food Anim. Pr..

[35] Olson M.E., Ralston B., Burwash L., Matheson-Bird H., Allan N.D. (2016). Efficacy of oral meloxicam suspension for prevention of pain and inflammation following band and surgical castration in calves. BMC Vet. Res..

[36] Sutherland M., Lowe G., Huddart F., Waas J., Stewart M. (2018). Measurement of dairy calf behavior prior to onset of clinical disease and in response to disbudding using automated calf feeders and accelerometers. J. Dairy Sci..

[37] Currah J.M., Hendrick S.H., Stookey J.M. (2009). The behavioral assessment and alleviation of pain associated with castration in beef calves treated with flunixin meglumine and caudal lidocaine epidural anesthesia with epinephrine. Can. Vet. J. Rev..

[38] Bates A., Eder P., Laven R., Laven R. (2015). Effect of analgesia and anti-inflammatory treatment on weight gain and milk intake of dairy calves after disbudding. N. Z. Vet. J..

[39] Mathews K.A. (2000). Pain assessment and general approach to management. Vet. Clin. N. Am. Small Anim. Pract..

[40] Williamson A., Hoggart B. (2005). Pain: A review of three commonly used pain rating scales. J. Clin. Nurs..

[41] Kielland C., Skjerve E., Zanella A.J. (2009). Attitudes of veterinary students to pain in cattle. Vet. Rec..

[42] Kielland C., Skjerve E., Østerås O., Zanella A.J. (2010). Dairy farmer attitudes and empathy toward animals are associated with Anim Welf indicators. J. Dairy Sci..

[43] Wikman I., Hokkanen A.-H., Pastell M., Kauppinen T., Valros A., Hänninen L. (2013). Dairy producer attitudes to pain in cattle in relation to disbudding calves. J. Dairy Sci..

[44] Mirra A., Spadavecchia C., Bruckmaier R., Gutzwiller A., Casoni D. (2018). Acute pain and peripheral sensitization following cautery disbudding in 1- and 4-week-old calves. Physiol. Behav..

[45] Laven R., Huxley J., Whay H., Stafford K. (2009). Results of a survey of attitudes of dairy veterinarians in New Zealand regarding painful procedures and conditions in cattle. N. Z. Vet. J..

[46] Tschoner T., Sauter-Louis C., Peinhofer V., Feist M. (2020). Attitudes of Bavarian bovine veterinarians towards pain and pain management in cattle. Vet. Rec..

[47] Weber C.N., Kerstin K.E. (2012). Schmerztherapie beim Rind-eine Umfrage unter der in der Rinderpraxis tätigen Tierärzten in Deuschland Teil II: Die Einschätztung von Schmerzen bei Rindern. Tiearztl. Umsch..

[48] Grisneaux E., Pibarot P., Dupuis J., Blais D. (1999). Comparison of ketoprofen and carprofen administered prior to orthopedic surgery for control of postoperative pain in dogs. J. Am. Vet. Med Assoc..

[49] Norring M., Wikman I., Hokkanen A.-H., Kujala M.V., Hänninen L. (2014). Empathic veterinarians score cattle pain higher. Vet. J..

[50] de Williams A.C.D.C. (2002). Facial expression of pain: An evolutionary account. Behav. Brain Sci..

[51] Müller B.R., Soriano V.S., Bellio J.C.B., Molento C.F.M. (2019). Facial expression of pain in Nellore and crossbred beef cattle. J. Vet. Behav..

[52] Gleerup K.B., Forkman B., Lindegaard C., Andersen P.H. (2015). An equine pain face. Vet. Anaesth. Analg..

[53] Tschoner T., Zablotski Y., Knubben-Schweizer G., Feist M. (2020). Xylazine application prior to laparoscopic abomasopexy in order to reduce stress in dairy cows. J. Dairy Sci..

[54] Rääf A., Olsen R.S. (2017). The Effect of Meloxicam Treatment after Disbudding on Pain-Related Behaviours and Weight Gain in Danish Holstein Calves. A Comparative Study between one day and four days of treatment. Master′s Thesis.

[55] Bamberg E., Wittke G. (1987). IX. Endokrinium. Lehrbuch der Veterinärphysiologie.

[56] Greco D., Stabenfeldt G.H., Cunningham J.G. (1997). Endocrine Glands and Their Function. Textbook of Veterinary Physiology.

[57] Hernandez C.E., Thierfelder T., Svennersten-Sjaunja K., Berg C., Orihuela A., Lidfors L. (2014). Time lag between peak concentrations of plasma and salivary cortisol following a stressful procedure in dairy cattle. Acta Vet. Scand..

[58] Pesenhofer G., Palme R., Pesenhofer R.M., Kofler J. (2006). Comparison of two methods of fixation during functional claw trimming-walk-in crush versus tilt table-in dairy cows using faecal cortisol metabolite concentrations and daily milk yield as parameters. Wien. Tierarztl. Monatsschr..

[59] Negrão J., Porcionato M., De Passillé A., Rushen J. (2004). Cortisol in Saliva and Plasma of Cattle After ACTH Administration and Milking. J. Dairy Sci..

[60] Gygax L., Neuffer I., Kaufmann C., Hauser R., Wechsler B. (2006). Milk Cortisol Concentration in Automatic Milking Systems Compared with Auto-Tandem Milking Parlors. J. Dairy Sci..

[61] Braun U., Michel N., Baumgartner M.R., Hässig M., Binz T.M. (2017). Cortisol concentration of regrown hair and hair from a previously unshorn area in dairy cows. Res. Vet. Sci..

[62] Stewart M., Webster J.R., Schaefer A.L., Cook N.J., Scott S.L. (2005). Infrared thermography as a non-invasive tool to study Animal Welfare. Anim. Welf..

[63] Heimbürge S., Kanitz E., Otten W. (2019). The use of hair cortisol for the assessment of stress in animals. Gen. Comp. Endocrinol..

[64] Comin A., Peric T., Magrin L., Corazzin M., Cornacchia G., Prandi A. (2014). Study of progesterone and cortisol concentrations in the Italian Friesian claw. J. Dairy Sci..

[65] Stock M., Millman S., Barth L., Van Engen N., Hsu W., Wang C., Gehring R., Parsons R., Coetzee J. (2015). The effects of firocoxib ojoachimn cautery disbudding pain and stress responses in preweaned dairy calves. J. Dairy Sci..

[66] Coetzee J.F., Gehring R., Tarus-Sang J., Anderson D.E. (2010). Effect of sub-anesthetic xylazine and ketamine (’ketamine stun’) administered to calves immediately prior to castration. Vet. Anaesth. Analg..

[67] Singh A.K., Jiang Y., White T., Spassova D. (1997). Validation of Nonradioactive Chemiluminescent Immunoassay Methods for the Analysis of Thyroxine and Cortisol in Blood Samples Obtained from Dogs, Cats, and Horses. J. Vet. Diagn. Investig..

[68] Russell N., Foster S., Clark P., Robertson I., Lewis D., Irwin P., Irwin P. (2007). Comparison of radioimmunoassay and chemiluminescent assay methods to estimate canine blood cortisol concentrations. Aust. Vet. J..

[69] Proverbio D., Perego R., Spada E., De Giorgi G.B., Belloli A.G., Pravettoni D. (2013). Comparison of VIDAS and Radioimmunoassay Methods for Measurement of Cortisol Concentration in Bovine Serum. Sci. World, J..

[70] Rongen H., Hoetelmans R., Bult A., Van Bennekom W. (1994). Chemiluminescence and immunoassays. J. Pharm. Biomed. Anal..

[71] Coetzee J.F., Edwards L.N., Mosher R.A., Bello N.M., O’Connor A.M., Wang B., KuKanich B., Blasi D.A. (2012). Effect of oral meloxicam on health and performance of beef steers relative to bulls castrated on arrival at the feedlot. J. Anim. Sci..

[72] Morisse J., Cotte J., Huonnic D. (1995). Effect of dehorning on behaviour and plasma cortisol responses in young calves. Appl. Anim. Behav. Sci..

[73] Heinrich A., Duffield T.F., Lissemore K.D., Squires E.J., Millman S.T. (2009). The impact of meloxicam on postsurgical stress associated with cautery dehorning. J. Dairy Sci..

[74] Bristow D.J., Holmes D.S. (2007). Cortisol levels and anxiety-related behaviors in cattle. Physiol. Behav..

[75] Ogino M., Matsuura A., Yamazaki A., Irimajiri M., Suzuki Y., Kushibiki S., Singu H., Kasuya E., Hasegawa Y., Hodate K. (2013). Plasma cortisol and prolactin secretion rhythms in cattle under varying external environments and management techniques. Anim. Sci. J..

[76] Tschoner T., Behrendt-Wipperman M., Rieger A., Metzner M., Knubben-Schweizer G., Reichmann F., Feist M. (2018). Course of plasma substance P concentrations during umbilical surgery in calves. Berl. Munch. Tierarztl. Wochenschr..

[77] Mayer C. (2019). Untersuchungen Zur Schmerzäußerung Bei Kälbern Nach Schwanzspitzenamputation. Ph.D. Thesis.

[78] Dockweiler J., Coetzee J., Edwards-Callaway L., Bello N., Glynn H., Allen K., Theurer M., Jones M., Miller K., Bergamasco L. (2013). Effect of castration method on neurohormonal and electroencephalographic stress indicators in Holstein calves of different ages. J. Dairy Sci..

[79] Mosher R.A., Coetzee J.F., Allen P.S., Havel J.A., Griffith G.R., Wang C. (2014). Effects of sample handling methods on substance P concentrations and immunoreactivity in bovine blood samples. Am. J. Vet. Res..

[80] Coetzee J.F., Mosher R.A., KuKanich B., Gehring R., Robert B., Reinbold J.B., White B.J. (2012). Pharmacokinetics and effect of intravenous meloxicam in weaned Holstein calves following scoop dehorning without local anesthesia. BMC Vet. Res..

[81] Hunt S.P., Mantyh P.W. (2001). The molecular dynamics of pain control. Nat. Rev. Neurosci..

[82] Mashaghi A., Marmalidou A., Tehrani M., Grace P.M., Pothoulakis C., Dana R. (2016). Neuropeptide substance P and the immune response. Cell. Mol. Life Sci..

[83] Geracioti T.D., Carpenter L.L., Owens M.J., Baker D.G., Ekhator N.N., Horn P.S., Strawn J.R., Sanacora G., Kinkead B., Price L.H. (2006). Elevated Cerebrospinal Fluid Substance P Concentrations in Posttraumatic Stress Disorder and Major Depression. Am. J. Psychiatry.

[84] Michelgård A., Appel L., Pissiota A., Frans O., Långström B., Bergström M., Fredrikson M. (2007). Symptom Provocation in Specific Phobia Affects the Substance P Neurokinin-1 Receptor System. Biol. Psychiatry.

[85] Joachim R.A., Hildebrandt M., Oder J., Klapp B.F., Arck P.C. (2001). Murine stress-triggered abortion is mediated by increase of CD8+ TNF-α+ decidual cells via substance P. Am. J. Reprod. Immunol..

[86] Tometten M., Klapp B.F., Joachim R., Fest S., Zenclussen A.C., Peters E.M., Hertwig K., Arck P.C. (2004). Nerve Growth Factor and its Functional Receptor TrkA are Up-regulated in Murine Decidual Tissue of Stress-triggered and Substance P-mediated Abortion. Am. J. Reprod. Immunol..

[87] Heinrich A., Duffield T., Lissemore K., Millman S. (2010). The effect of meloxicam on behavior and pain sensitivity of dairy calves following cautery dehorning with a local anesthetic. J. Dairy Sci..

[88] White B.J., Anderson D.E., DuCharme A., Miesner M.D., Larson R.L., Amrine D. (2013). Multimodal assessment of biometric changes in injection sites and physiology and behavior in beef calves receiving two different clostridial immunizations compared to negative controls. Int. J. Appl. Vet. Med..

[89] Casoni D., Mirra A., Suter M., Gutzwiller A., Spadavecchia C. (2019). Can disbudding of calves (one versus four weeks of age) induce chronic pain?. Physiol. Behav..

[90] Hartnack A.K. (2014). Use of Analgesic Combination Morphine-Lidocaine-Ketamine in Holstein Calves Undergoing Ventral Midline Herniorrhaphy. Ph.D. Thesis.

[91] Harris L., Murrell J., Van Klink E., Whay H. (2015). Influence of experimental protocol on response rate and repeatability of mechanical threshold testing in dogs. Vet. J..

[92] White B.J., Coetzee J.F., Renter D.G., Babcock A.H., Thomson D.U., Andresen D. (2008). Evaluation of two-dimensional accelerometers to monitor behavior of beef calves after castration. Am. J. Vet. Res..

[93] Ravi N., Dandekar N., Mysore P., Littman M.L. (2005). Activity recognition from accelerometer data. Aaai.

[94] Costa J.H., Cantor M.C., Neave H.W. (2021). Symposium review: Precision technologies for dairy calves and management applications. J. Dairy Sci..

[95] Pauly C., White B.J., Coetzee J.F., Robert B., Baldridge S., Renter D.G. (2012). Evaluation of analgesic protocol effect on calf behavior after concurrent castration and dehorning. Int. J. Appl. Res. Vet. Med..

[96] Theurer M., White B.J., Coetzee J.F., Edwards L.N., Mosher R.A., Cull C.A. (2012). Assessment of behavioral changes associated with oral meloxicam administration at time of dehorning in calves using a remote triangulation device and accelerometers. BMC Vet. Res..

[97] Rushen J., Chapinal N., De Passille A.M. (2012). Automated monitoring of behavioural-based animal welfare indicators. Anim. Welf. UFAW J..

[98] Theurer M.E., Amrine D.E., White B.J. (2013). Remote Noninvasive Assessment of Pain and Health Status in Cattle. Vet. Clin. North Am. Food Anim. Pract..

[99] Pieler D., Peinhopf W., Becher A., Aurich J., Rose-Meierhöfer S., Erber R., Möstl E., Aurich C. (2013). Physiological and behavioral stress parameters in calves in response to partial scrotal resection, orchidectomy, and Burdizzo castration. J. Dairy Sci..

[100] Coetzee J.F., Mosher R.A., Anderson D.E., Robert B., Kohake L.E., Gehring R., White B.J., KuKanich B., Wang C. (2014). Impact of oral meloxicam administered alone or in combination with gabapentin on experimentally induced lameness in beef calves. J. Anim. Sci..

[101] Musk G.C., Jacobsen S., Hyndman T.H., Lehmann H.S., Tuke S.J., Collins T., Gleerup K.B., Johnson C.B., Laurence M. (2017). Objective Measures for the Assessment of Post-Operative Pain in Bos indicus Bull Calves Following Castration. Animals.

[102] Robért B.D., White B.J., Renter D.G., Larson R.L. (2011). Determination of lying behavior patterns in healthy beef cattle by use of wireless accelerometers. Am. J. Vet. Res..

[103] Haley D.B., Bailey D.W., Stookey J.M. (2005). The effects of weaning beef calves in two stages on their behavior and growth rate. J. Anim. Sci..

[104] Stelletta C., Gianesella M., Vencato J., Fiore E., Morgante M., Prakash R. (2012). Thermographic applications in veterinary medicine. Infrared Thermography.

[105] Metzner M., Sauter-Louis C., Seemueller A., Petzl W., Zerbe H. (2015). Infrared thermography of the udder after experimentally induced Escherichia coli mastitis in cows. Vet. J..

[106] Berry R.J., Kennedy A.D., Scott S.L., Kyle B.L., Schaefer A.L. (2003). Daily variation in the udder surface temperature of dairy cows measured by infrared thermography: Potential for mastitis detection. Can. J. Anim. Sci..

[107] Harris-Bridge G., Young L., Handel I., Farish M., Mason C., Mitchell M., Haskell M. (2018). The use of infrared thermography for detecting digital dermatitis in dairy cattle: What is the best measure of temperature and foot location to use?. Vet. J..

[108] McManus C., Tanure C.B., Peripolli V., Seixas L., Fischer V., Gabbi A.M., Menegassi S.R., Stumpf M.T., Kolling G.J., Dias E. (2016). Infrared thermography in animal production: An overview. Comput. Electron. Agric..

[109] Stewart M., Stookey J., Stafford K., Tucker C., Rogers A., Dowling S., Verkerk G., Schaefer A., Webster J. (2009). Effects of local anesthetic and a nonsteroidal antiinflammatory drug on pain responses of dairy calves to hot-iron dehorning. J. Dairy Sci..

[110] Stewart M., Stafford K., Dowling S., Schaefer A., Webster J. (2008). Eye temperature and heart rate variability of calves disbudded with or without local anaesthetic. Physiol. Behav..

[111] Stewart M., Verkerk G., Stafford K., Schaefer A., Webster J. (2010). Noninvasive assessment of autonomic activity for evaluation of pain in calves, using surgical castration as a model. J. Dairy Sci..

[112] Stewart M., Schaefer A.L., Haley D.B., Colyn J., Cook N.J., Stafford K.J., Webster J.R. (2008). Infrared thermography as a non-invasive method for detecting fear-related responses of cattle to handling procedures. Anim. Welf..

[113] Schaefer A.L., Cook N.J., Tessaro S.V., Deregt D., Desroches G., Dubeski P.L., Tong A.K., Godson D.L. (2004). Early detection and prediction of infection using infrared thermography. Can. J. Anim. Sci..

[114] Metzner M., Sauter-Louis C., Seemueller A., Petzl W., Klee W. (2014). Infrared thermography of the udder surface of dairy cattle: Characteristics, methods, and correlation with rectal temperature. Vet. J..

[115] Jimenez R.E., Adcock S.J., Tucker C.B. (2019). Acute pain responses in dairy calves undergoing cornual nerve blocks with or without topical anesthetic. J. Dairy Sci..

[116] von Borell E., Langbein J., Després G., Hansen S., Leterrier C., Marchant-Forde J., Marchant-Forde R., Minero M., Mohr E., Prunier A. (2007). Heart rate variability as a measure of autonomic regulation of cardiac activity for assessing stress and welfare in farm animals—A review. Physiol. Behav..

[117] Byrd C.J., Craig B.A., Eicher S.D., Radcliffe J.S., Lay Jr D.C. (2019). Assessment of disbudding pain in dairy calves using nonlinear measures of heart rate variability. J. Dairy Sci..

[118] Adcock S.J.J., Tucker C.B. (2020). The effect of early burn injury on sensitivity to future painful stimuli in dairy heifers. PLoS ONE.

[119] Mohr E., Langbein J., Nürnberg G. (2002). Heart rate variability: A noninvasive approach to measure stress in calves and cows. Physiol. Behav..

[120] Braun U., Zürcher S., Hässig M. (2015). Evaluation of eating and rumination behaviour in 300 cows of three different breeds using a noseband pressure sensor. BMC Vet. Res..

[121] Braun U., Tschoner T., Hässig M., Nuss K. (2017). Eating and rumination behaviour in cows with traumatic reticuloperitonitis. Schweiz Arch Tierheilkd.

[122] Eslamizad M., Tümmler L.-M., Derno M., Hoch M., Kuhla B. (2018). TECHNICAL NOTE: Development of a pressure sensor-based system for measuring rumination time in pre-weaned dairy calves. J. Anim. Sci..

[123] Swartz T., Findlay A., Petersson-Wolfe C. (2017). Short communication: Automated detection of behavioral changes from respiratory disease in pre-weaned calves. J. Dairy Sci..

[124] González L.A., Schwartzkopf-Genswein K.S., Caulkett N.A., Janzén E., McAllister T.A., Fierheller E., Schaefer A.L., Haley D.B., Stookey J.M., Hendrick S. (2010). Pain mitigation after band castration of beef calves and its effects on performance, behavior, Escherichia coli, and salivary cortisol. J. Anim. Sci..

[125] Repenning P.E., Ahola J.K., Callan R.J., French J.T., Giles R.L., Bigler B.J., Coetzee J.F., Wulf L.W., Peel R.K., Whittier J.C. (2013). Impact of oral meloxicam administration before and after band castration on feedlot performance and behavioral response in weanling beef bulls. J. Anim. Sci..

[126] Schwartzkopf-Genswein K.S., Stookey J.M., Janzen E.D., McKinnon J. (1997). Effects of branding on weight gain, antibiotic treatment rates and subsequent handling ease in feedlot cattle. Can. J. Anim. Sci..

[127] McMeekan C., Stafford K., Mellor D., Bruce R., Ward R., Gregory N. (1998). Effects of regional analgesia and/or a non-steroidal anti-inflammatory analgesic on the acute cortisol response to dehorning in calves. Res. Vet. Sci..

[128] Eicher S.D., Dailey J.W. (2002). Indicators of Acute Pain and Fly Avoidance Behaviors in Holstein Calves Following Tail-docking. J. Dairy Sci..

[129] Sutherland M., Mellor D., Stafford K., Gregory N., Bruce R., Ward R. (2002). Cortisol responses to dehorning of calves given a 5-h local anaesthetic regimen plus phenylbutazone, ketoprofen, or adrenocorticotropic hormone prior to dehorning. Res. Vet. Sci..

[130] Mintline E.M., Varga A., Banuelos J., Walker K.A., Hoar B., Drake D., Weary D., Coetzee J.F., Stock M.L., Tucker C.B. (2014). Healing of surgical castration wounds: A description and an evaluation of flunixin. J. Anim. Sci..

[131] McCarthy D.R., Lomax S., Windsor P., White P. (2016). Effect of a topical anaesthetic formulation on the cortisol response to surgical castration of unweaned beef calves. Animal.

[132] Adcock S.J., Tucker C.B. (2018). The effect of disbudding age on healing and pain sensitivity in dairy calves. J. Dairy Sci..

[133] Park S.J., Piao M., Kim H., Kang H.J., Seo J., Lee S., Baik M. (2018). Effects of castration and a lidocaine-plus-flunixin treatment on growth and indicators of pain, inflammation, and liver function in Korean cattle bull calves. Livest. Sci..

[134] Cuttance E.L., Mason W.A., Yang D.A., Laven R.A., McDermott J., Inglis K. (2019). Effects of a topically applied anaesthetic on the behaviour, pain sensitivity and weight gain of dairy calves following thermocautery disbudding with a local anaesthetic. N. Z. Vet. J..

[135] Adcock S.J., Cruz D.M., Tucker C.B. (2020). Behavioral changes in calves 11 days after cautery disbudding: Effect of local anesthesia. J. Dairy Sci..

[136] Adcock S.J.J., Tucker C.B. (2020). Conditioned place preference reveals ongoing pain in calves 3 weeks after disbudding. Sci. Rep..

